# Concept Maps to Assess System Understanding: Are Graphical Explanations More Accurate than Verbal Ones?

**DOI:** 10.3390/bs14090807

**Published:** 2024-09-11

**Authors:** Judith Schmidt, Lilli Wollermann, Stephan Abele, Romy Müller

**Affiliations:** 1 Faculty of Education, TUD Dresden University of Technology, 01069 Dresden, Germany; stephan.abele@tu-dresden.de; 2 Faculty of Psychology, TUD Dresden University of Technology, 01069 Dresden, Germanyromy.mueller@tu-dresden.de (R.M.)

**Keywords:** functional system understanding, functional abstraction hierarchy, concept mapping, assessment method, mental representation

## Abstract

Solving problems in a technical system usually requires people to understand its functioning on different levels of abstraction (i.e., goals, functions, components, characteristics) that are connected via means–ends links. We combined this abstraction hierarchy with concept mapping to assess people’s understanding of system functioning. The present study examines the benefits and drawbacks of the proposed method by comparing it to a viable alternative, namely verbal explanation. Using a set of pre-defined concepts, twenty-four participants explained the functioning of two everyday systems: one graphically by constructing a concept map and one verbally. The verbal explanations were subsequently transformed into concept maps by the authors. Compared to verbal explanations, participant-constructed concept maps contained a higher proportion of functional propositions, and lower proportions of structural, temporal, general, and other propositions. Contrary to our expectations, there was no difference regarding the accuracy of functional propositions. Even though participants needed far less time to explain system functioning verbally, our results indicate that concept mapping is better suited to assess functional system understanding. We discuss how this benefit relates to the cognitive processes during concept mapping, and how the method needs to be adapted to assess functional understanding of more complex systems.

## 1. Introduction and Theoretical Background

Solving problems that occur in a technical system usually requires people to understand that system’s functioning. How a system functions can be described on different levels of functional abstraction representing the purposes, functions, components, and properties of the system [1]. Consider the example of a simple bike. Here, the goal is to move from A to B (purpose), and this is typically achieved by pedaling. How does pedaling cause the wheels to turn? The pedals are connected to a chain ring (component) which transmits the turning motion (function) from the pedals onto the chain, and from there onto the back wheel (components). Understanding how the motion transmission works may not be necessary for riding your bike (i.e., for operating the system). It may be sufficient to know that when you pedal, the wheels are put into motion and this allows you to achieve your goal. However, if the system does not work properly, diagnosing and fixing the problem may require an understanding of the function that each component realizes, and how these functions work together to achieve the system’s purpose.

Previous research highlights the importance of functional knowledge for problem solving. For example, experts use functional principles to represent their knowledge about a system [2,3] and to assess the similarity of different problems [4]. Informing participants about functional principles also leads to better diagnostic performance than informing them about diagnostic procedures, especially when the diagnostic problems are complex [5]. Thus, having a correct functional understanding seems to be an important prerequisite for successfully solving problems within that system. To assess whether people know how a system functions, methods are needed that specifically address functional understanding. Functional understanding can be assessed by having learners create some form of graphical representation of the system. One way of doing this is to ask them to draw a concept map specifying the system functions and how they are realized. While graphical representations in general and concept maps in particular have previously been used to assess understanding (see [6] for a review), they are rarely constructed according to an overarching structural principle. The present study examines the following questions: Can a combination of concept mapping and a specific functional framework (i.e., the abstraction hierarchy) be used to assess functional understanding? What benefits and drawbacks arise compared to explaining system functioning verbally?

### 1.1. Representing System Functioning on Different Levels of Abstraction

System functioning can be conceptualized in several different ways (e.g., [1,7,8,9]). In the present study, it is defined according to Rasmussen’s abstraction hierarchy [1]. This hierarchy describes a system on different levels of functional abstraction that are connected by means–ends relations. More precisely, the higher levels explain why the lower levels are necessary, and the lower levels explain how the higher levels are realized. On the top level, the *functional purpose* or goal of the system is described. Returning to the example of a bike, the goal is to move around. How this goal is achieved is described on the next level of higher-order *abstract functions.* For example, riding a bike requires the wheels to turn. The next level contains *generalized functions* that show how the abstract functions are realized. To get the wheels to turn, the motion must be transmitted onto the wheels. The next level describes the *physical functions* or components realizing these functions, such as the pedals or the chain for motion transmission. Finally, the lowest level describes the *physical form*, namely the specific properties that components need for carrying out their respective functions. For example, different sprocket sizes are used to modulate the motion transmission. While these five levels have proven useful for describing different types of systems, the exact number of levels can be adapted to the complexity of the respective system and the level of detail that is required [10].

The abstraction hierarchy has frequently been used to enhance the interaction between people and technical systems by providing information on certain levels of abstraction. Empirical studies have shown that when people take information from all levels into account, they are in fact better able to solve problems [11,12] and need less time to identify critical aspects within a situation [13]. However, the information is not used separately, but integrated across the levels of abstraction [14]. If the means–ends relations between higher and lower levels of abstraction are made explicit, participants seem to come up with more solutions for solving a problem within the system [15]. These studies support the notion that when interacting with a technical system, people use and integrate information from different levels of abstraction. It is, however, not clear whether they use these levels and the means–ends links between them to internally represent their system knowledge. By combining the abstraction hierarchy with a graphical representation method, we can examine whether participants are able to accurately express their understanding of system functioning. One promising method in this regard is a particular form of graphical representation, namely concept mapping.

### 1.2. Concept Maps to Assess Understanding of System Functioning

Concept maps are directed graphs in which the concepts are represented as nodes, and the relations between these concepts are represented as labeled arrows. Consequently, these graphs can be used to externalize the content and structure of knowledge [16,17,18]. Since concept maps can be successfully constructed by people with varying spatial and verbal abilities [19,20,21], they appear to be suitable for assessing understanding of system functioning.

Concept mapping tasks can afford different levels of directedness [22]. Low directedness can be achieved by only providing the topic of the concept map, thereby requiring people to choose which concepts to include in their map and how to relate them to each other [23]. This leads to high variance and difficulties when trying to compare concept maps that were constructed by different people. High directedness, on the other hand, can be achieved by providing people with a structure and labeled links as well as a list of concepts to use, so that they only have to fill in the gaps [24,25,26]. Such highly directed concept mapping tasks do, however, restrict people from expressing their internal understanding. Thus, concept maps for which only the concepts have been provided are better able to represent differences in understanding between participants [27,28]. In studies that ask participants to construct a concept map about their understanding, their task typically is to include all concepts and relations that they perceive to be relevant. Thus, neither the maps constructed by participants nor those used as reference structures follow a specific overarching principle. Instead, a single map often contains different types of information, such as general and part–whole relations between concepts, temporal relations, examples, or information about causal relations and consequences. From a practical standpoint, the lack of an overarching principle means that educators and researchers typically construct a new reference map for their respective topic from scratch, often by consulting multiple experts. However, experts may produce different representations about the same topic [16,29], entailing the question of which of these representations is most suitable. From a theoretical standpoint, the lack of an overarching principle makes it harder to draw conclusions across topics. Applying a framework like the abstraction hierarchy could facilitate the transfer of results between studies, independent of the specific topic in focus.

To combine concept mapping with the abstraction hierarchy, the contents of the respective abstraction levels could be provided as nodes (i.e., the specific purposes, functions, components, and properties). Participants would then be asked to generate and describe the means–ends relations (i.e., functional relations) between these concepts. The accuracy of the resulting concept maps can easily be compared to a reference map, for example, by looking at the propositions within each map [30]. Propositions are the smallest meaningful component of a concept map that consist of two concepts and the relation between them (e.g., movement transmission—is realized by—chain). Examining these propositions enables conclusions about the quality of a concept map and consequently about participants’ understanding of system functioning.

Despite the obvious potentials of concept mapping, it has previously been mentioned that this technique requires practice [21,31]. A low-quality concept map may therefore indicate a low understanding of system functioning, but it may also indicate problems in using the method. When constructing a concept map, one must specify the functional relations between concepts and plan the construction process (e.g., where to place concepts [19] and what links to include [32]), but this is by no means trivial [33]. If the structure of the concept map remains unclear, participants may feel disoriented and report higher levels of cognitive load [34], which may impair their performance during the mapping task. Thus, being able to structure one’s understanding of system functioning is an important prerequisite for constructing an accurate graphical representation. If participants are not able to meet these method-related requirements, their actual system understanding will likely be underestimated. To mitigate such problems, participants could also explain system functioning verbally, and for further analysis, this explanation could subsequently be transformed into a concept map by someone familiar with the construction of concept maps. Which of these two elicitation methods should be used to assess participants’ understanding of system functioning?

### 1.3. Present Study

Is concept mapping a valid method to assess people’s understanding of system functioning? What benefits and drawbacks arise when people use concept mapping compared to when they can express their understanding verbally? To answer these questions, we asked participants to use both methods for explaining how two everyday systems function, namely a bike and a sink. All participants were introduced to the abstraction hierarchy and how it can be used to describe system functioning. The accuracy of participants’ understanding was determined through comparison with a reference map. Since both systems used in the present study are well known, we assumed that all participants had a perfect understanding of system functioning, at least regarding the depth that was required here. Thus, the structure-related and content-related quality of participants’ explanations was interpreted as an indicator for the validity of the respective method.

Based on the degrees of freedom that concept mapping and verbal explanation afford, we expected differences regarding the structure-related quality. When constructing a concept map, one must actively decide where to put each concept so that the concept map structure represents how the concepts are related to each other [19]. Verbal explanations, on the other hand, are much more flexible and spontaneous. We therefore expected a higher structure-related quality of participants’ graphical explanations. We also expected differences regarding the content-related quality. To achieve high content-related quality, participants must first use functional relations. While neither concept mapping nor verbal explanations have an inherent focus on functional relations, we expected that participants would be more conscious of this requirement during concept mapping compared to verbal explanation. Consequently, we expected graphical explanations to contain more functional propositions. Second, participants need to provide accurate descriptions of the functional relations between concepts to construct concept maps with high content-related quality. Here, we expected the flexibility afforded by verbal explanations to be beneficial. If participants know how a system functions, they should be able to describe it in their own words. Concept mapping is a more formalized approach and thus, formal mistakes are possible (e.g., mixing up the direction of the arrow that connects two concepts). In addition, another study in our lab raised the suspicion that participants might be inclined to adopt the terminology used in the abstraction hierarchy (e.g., *is realized by* as a description of a means–ends relation), even if it is not an appropriate description of the relation between the two concepts in question. Therefore, we expected graphical explanations to contain fewer accurate functional propositions. The present article thus makes the following contributions:

- We propose a new method for explaining system functioning, namely concept mapping following the structure of a functional abstraction hierarchy.
- We describe an empirical study examining whether these concept maps enable valid assessments of functional system understanding and derive recommendations for the future use of the concept mapping method.
- To facilitate the future development of the method and its application in other contexts, all materials, data, and analyses were made available via the Open Science Framework (https://osf.io/kneah/).

## 2. Method

### 2.1. Participants

Twenty-four participants (fifteen female, nine male) were recruited via the TUD Dresden University of Technology’s participant pool. Their age ranged from 19 to 71 years (*M* = 28.4, *SD* = 13.0). As the systems used in the study are very common, the only inclusion criterion was understanding German and speaking it fluently. All procedures followed the principles of the Declaration of Helsinki.

### 2.2. Apparatus and Stimuli

#### 2.2.1. Technical Setup

Participants were invited to a lab at the university, where two of the authors acted as experimenters: LW was physically present and RM joined virtually via Zoom. Zoom was also used to record the experimental session with participants’ consent. A laptop (15.6″ screen) was used to present all materials, and to carry out the graphical representation task. Concept maps were constructed with CmapTools (Version 6.04, Florida Institute for Human and Machine Cognition, 2019).

#### 2.2.2. Instruction Videos

The instructions consisted of three videos (each between four and six minutes long) that explained the functional abstraction hierarchy, concept map construction, and different types of functional relations, respectively. All videos were presented in German. The first video introduced the functional abstraction hierarchy as consisting of different levels to represent the goals, functions, components, and properties of a system, and of the means–ends relations between these levels. The abstraction hierarchy was illustrated using one simplified aspect of a flying aircraft (i.e., that in order to fly, an aircraft requires thrust and lift, which is ultimately realized by the engine and the curved form of the wings). The video also explained that for each given level of abstraction, the lower level specifies how a function is implemented, and the higher level specifies why that function is required. The second video explained how participants should construct their concept maps using CmapTools. Participants were told that they were not allowed to add concepts and that if they did, these concepts would be deleted prior to data analysis. They were encouraged to start with the goal and then proceed with the functions, components, and properties. At the end of the second video, the experimental task was specified. Participants were asked to use verbs for specifying the relation between two concepts, and to describe these relations as precisely as possible. The third video focused on connecting concepts via arrows and labeling these arrows according to the relations between the concepts. This was achieved by explaining the different types of functional relations used in the example concept map (realizes/is realized by, has a precondition/is a precondition for, and has a property/is a property of). Participants were informed that these descriptions were only examples and they were encouraged to use descriptions that best explained the functional relation between two concepts as they understood it. The importance of matching the direction of the arrow to the relation description was also highlighted.

#### 2.2.3. Reference Maps and Provided Materials

**Figure 1:**
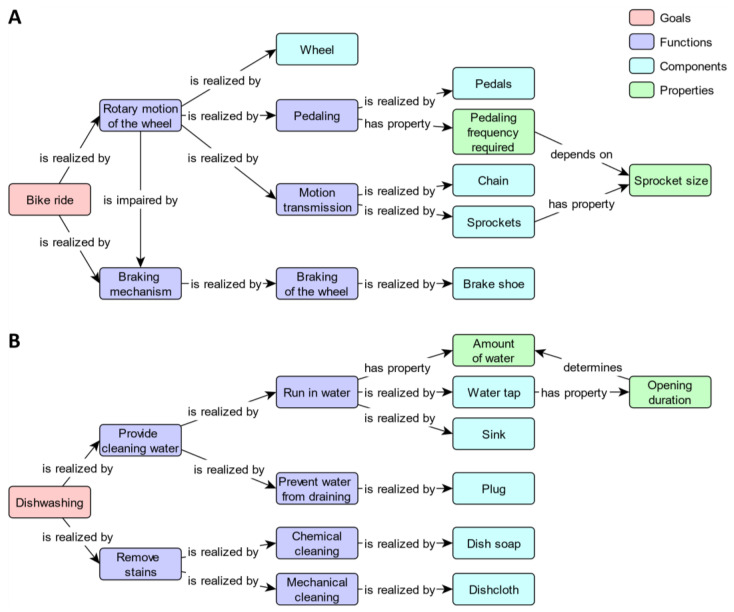
Reference maps for (**A**) bike and (**B**) sink.

To evaluate participants’ explanations, a reference map was constructed for each topic prior to data collection. Both reference maps are depicted in Figure 1. As can be seen, these maps included only correct functional propositions and followed the abstraction hierarchy structure. The reference maps were not shown to participants.

To explain their understanding of system functioning, participants were provided with a fixed set of concepts: 13 concepts for explaining the bike and 14 concepts for explaining the sink. As system functioning could be explained with 14 propositions for both topics, we assumed similar levels of complexity. Participants were not allowed to add concepts, but could omit those for which they did not know how they contributed to system functioning. In the graphical representation condition, the concepts were provided directly in CmapTools. They were placed in the top part of the screen in an unconnected and unordered way. In the verbal explanation condition, the concepts were presented in the same way, but as a screenshot to prevent participants from interacting with the material (e.g., by sorting or rearranging concepts). For both methods and in accordance with the instruction videos, the color of each concept indicated whether it represented a goal, function, component, or property.

### 2.3. Procedure

**Figure 2:**
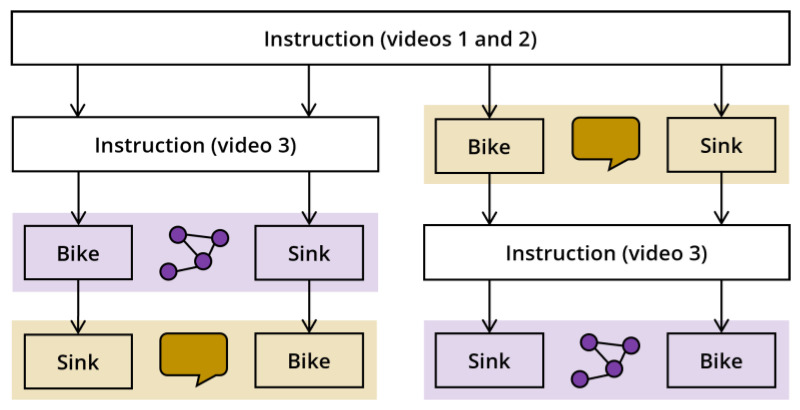
Overview of the study procedure. Purple highlights represent the graphical representation condition, and yellow highlights represent the verbal explanation condition.

In a within-subjects design, each participant explained the functioning of the bike and the sink, one by concept mapping and the other by verbal explanation. The order of assessment methods and the combination of assessment methods and topics were counterbalanced across participants, resulting in four groups that participants were randomly assigned to. Figure 2 presents an overview of the study procedure.

The experimental procedure started with the first instruction video. Participants then had the opportunity to ask questions and were subsequently asked to read the example map used in the instruction video (flying aircraft) out loud. This exercise was included to ensure that all participants had understood the general structure of a concept map and knew that the direction of the arrow between two concepts specified the direction of how the relation was to be read. Afterwards, the second video was shown and participants were again able to ask questions.

For those participants starting with the graphical representation task (n = 12), the third instruction video was shown next. Participants were subsequently asked to use the provided concepts to construct a concept map about either the bike or the sink. They were allowed to ask questions in case of any difficulties with using the software. Questions about system functioning were not answered. The verbal explanation task followed, in which participants were asked to explain the other system’s functioning (bike or sink), again using the provided concepts. Participants were given time to think about their explanation and were asked to inform LM when they were ready to start. They were also informed that while they explained system functioning verbally, RM would construct a concept map and ask follow-up questions if some relations had remained unclear. More specifically, if participants had not clearly indicated which concepts they were referring to, RM restated the relation description and asked about the concepts. However, no follow-up questions were asked about the relations themselves.

The other participants (n = 12) started with the verbal explanation task. Afterwards, the third instruction video was shown and participants proceeded with the concept mapping task. Overall, the experiment took about 45 to 60 min.

### 2.4. Data Processing and Analysis

#### 2.4.1. Transforming Verbal Explanations into Concept Maps

To compare the representations of system functioning from both assessment methods, the verbal explanations were transformed into concept maps by RM. A first draft was constructed during the experiment. Based on the recordings, RM then transformed each participant’s explanation into a complete concept map. The verbal expressions (i.e., phrases or entire sentences) were fully transferred onto the concept map. No reductions, interpretations, or standardizations were conducted. To make sure that all information was retained, JS then cross-checked all concept maps using the experiment recordings.

#### 2.4.2. Dependent Variables

*Time on task* was recorded to identify the time expenditure associated with graphical representation and verbal explanation. In both conditions, time on task started once participants had received all instructions. In the graphical representation condition, time on task ended once participants stated that they had completed their concept map. In the verbal explanation condition, time on task ended once participants stated that they had finished their explanation or after they had answered any follow-up questions from RM.

To indicate the size of each concept map, the number of *propositions* was determined. Each proposition consisted of two concepts and the relation description.

**Figure 3:**
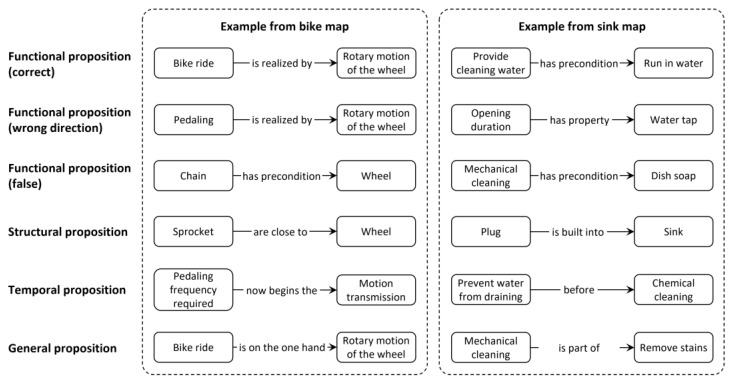
Example propositions for each proposition type and concept map topic.

The *proposition type* was categorized as functional, structural, temporal, general, or other. Thus, each proposition (i.e., each concept–relation–concept unit) was categorized as a functional, structural, temporal, general, or other type of statement. The functional, structural, and temporal categories were established prior to data analysis, whereas the category of general connectedness emerged as a new category from the “other” category. As the present research was specifically interested in participants’ functional understanding, all functional propositions were differentiated further into three *functional proposition types*, depending on their accuracy. Functional propositions were coded as “correct” if the functional relation established between the two connected concepts was true. The category “wrong direction” was assigned if the proposition would have been correct, had the direction of the linking arrow been reversed. The category “false” was assigned if a functional relation was used to connect two concepts that are not functionally related, or if the functional connection was not described correctly. These categories were established prior to data analysis. To give a better impression of the different proposition types, Figure 3 provides one example for each proposition type and map.

To assess the structure-related quality of each concept map (i.e., how closely it follows the structure of the abstraction hierarchy), the *intersection over union* (cf. [35]) was calculated. This measure represents the ratio of connections found in both the participant’s and the reference map and the sum of all connections in either the reference map or the participant’s map. Thus, a participant map including all connections from the reference map and no additional connections (i.e., only true positives and true negatives) would receive the maximum value of 1. Deviations from the maximum value arose if participants did not include connections from the reference map (i.e., false negatives) and if they added connections that were not part of the reference map (i.e., false positives). A participant map including none of the connections in the reference map (i.e., only false positives and false negatives) would receive the minimum value of 0. Within the material for explaining the bike ride, there was ambiguity regarding the ordering of two concepts. More specifically, both “braking mechanism” and “braking of the wheel” could be interpreted as describing a more abstract function than the other. Consequently, for both of these concepts, connections to the more abstract purpose as well as more concrete components were accepted as correct.

#### 2.4.3. Statistical Analyses

All analyses were conducted with IBM SPSS Statistics (Version 28). We conducted paired-samples *t*-tests to compare graphical representations and verbal explanations with regard to time on task, propositions, correct functional propositions, and intersection over union. To test whether the two assessment methods differed regarding the occurrence of certain proposition types, we conducted a 2 (*assessment method*: *graphical representation*, *verbal explanation*) × 5 (*proposition type*: *functional*, *structural*, *temporal*, *general*, *other*) repeated-measures ANOVA. To test whether the two assessment methods differed regarding the occurrence of certain functional proposition types, we conducted a 2 (*assessment method*: *graphical representation*, *verbal explanation*) × 3 (*functional proposition type*: *correct*, *wrong direction*, *inappropriate function*) repeated-measures ANOVA. The Greenhouse–Geisser adjustment was used to correct for violations of sphericity and the degrees of freedom were adjusted accordingly. If not otherwise specified, data from all 24 participants were available for analysis. We also checked whether the randomization procedure worked by testing for influences of the order of conditions as well as the combination of the system and assessment method. Since these analyses did not indicate significant influences, we can assume that the results did not differ for the first and second concept maps, and that both systems were similar in their level of complexity.

## 3. Results

### 3.1. Time on Task

For analyzing time on task, data from two participants were excluded. For one participant, the verbal explanation had not been recorded properly and thus the data were not reliable. The other participant took over twice as long to complete the graphical representation task compared to the mean of all other participants and thus this participant was classified as an outlier. Time on task was much higher for graphical representation than verbal explanation (17:07 vs. 3:47 min:s, respectively), *t*(21) = 11.59, *p* < 0.001, *d* = 2.47 (see Table 1). Thus, participants took over four times as long when explaining system functioning graphically compared to verbally.

### 3.2. Propositions and Correct Functional Propositions

**Figure 4:**
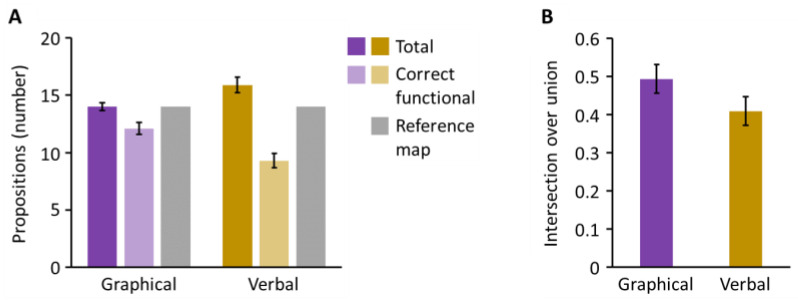
(**A**) Propositions and correct functional propositions, and (**B**) structural alignment with the reference map for graphical representation and verbal explanation.

The number of propositions was lower for graphical representation than for verbal explanation (14.0 vs. 15.9, respectively), *t*(23) = −2.82, *p* = 0.010, *d* = −0.58. However, the number of correct functional propositions was higher for graphical representation than for verbal explanation (12.1 vs. 9.3, respectively), *t*(23) = 3.87, *p* < 0.001, *d* = 0.79 (see Table 2). As can be seen in Figure 4A, participants used fewer propositions when explaining system functioning graphically, but included a higher number of correct functional propositions.

### 3.3. Proposition Types

Since the overall number of propositions differed between graphical representation and verbal explanation, proportions were used for analyzing the use of different proposition types. For both assessment methods, the proportions summed up to 100, so the main effect on the assessment method was not interpretable and therefore not reported. The analysis revealed a significant main effect of proposition type, *F*(1.87, 42.97) = 449.1, *p* < 0.001, η^2^ = 0.95. Pairwise comparisons indicated that irrespective of the assessment method, participants used a higher proportion of functional propositions than structural, temporal, general, and other propositions, all *p*s < 0.001. Participants also used a higher proportion of general than temporal propositions, *p* = 0.006, and other propositions, *p* < 0.001 (see Table 2 for mean values). Thus, functional propositions were generally most prevalent in participants’ explanations. In addition, a significant interaction between the assessment method and proposition type was found, *F*(2.24, 51.54) = 53.4, *p* < 0.001, η^2^ = 0.70. Pairwise comparisons indicated that compared to verbal explanations, graphical representations contained a higher proportion of functional propositions, *p* < 0.001, and a lower proportion of structural, temporal, general, and other propositions, all *p*s < 0.023 (see Table 2 for mean values). As hypothesized, participants used more functional and fewer non-functional propositions when explaining system functioning graphically.

A closer look into the quality of the functional propositions revealed a significant main effect of functional proposition type, *F*(1.33, 30.63) = 841.7, *p* < 0.001, η^2^ = 0.97. Pairwise comparisons indicated that irrespective of the assessment method, participants reported a higher proportion of correct functional propositions than propositions with a wrong direction or false functional propositions, all *p*s < 0.001 (see Table 2 for mean values). There was no significant interaction between the assessment method and functional proposition type, *F*(1.29, 29.72) = 2.61, *p* = 0.109. Contrary to our expectations, the proportion of different functional proposition types did not depend on which assessment method was used.

### 3.4. Intersection over Union

For the intersection over union, the difference between graphical representation and verbal explanation (0.49 vs. 0.41, respectively) missed the significance level, *t*(23) = 1.60, *p* = 0.061, *d* = 0.33. While Figure 4 suggests a trend towards higher structural alignment between participants’ maps and the reference map, participants did not follow the abstraction hierarchy significantly more closely when explaining system functioning with concept maps.

## 4. Discussion

What benefits and drawbacks arise when participants use concept mapping to explain how a system works compared to when they simply explain it verbally? We addressed this question by comparing concept mapping to a viable alternative for assessment, namely verbal explanation. More specifically, participants were asked to explain the functioning of two everyday systems (bike and sink) according to an abstraction hierarchy, one by using graphical representation and the other by using verbal explanation. We examined whether the structure-related and content-related concept map quality (i.e., similarity to a reference map) differed between both assessment methods.

Contrary to our expectation, the intersection over union (i.e., the structural similarity to the reference map) was not higher for concept mapping. While we observed a descriptive trend in the hypothesized direction, this difference may have been too small to be detected with our sample size. However, it is also possible that a purely structural (i.e., content-free) measure is not adequate to assess functional understanding. The intersection over union quantifies whether the relations included in a reference map are also included in a participant’s map. In the present study, the reference map was constructed according to the abstraction hierarchy and thus, the intersection over union decreased not only if participants added or omitted relations, but also if they changed the order of the abstraction levels. When we used this concept mapping method in a study with automotive technician apprentices [36], we consulted domain experts who felt that the particular order of levels in the abstraction hierarchy did not represent system functioning optimally in the context of automotive fault diagnosis and repair. Even though they would have chosen a different structure, they accurately explained system functioning. In that case, a small intersection over union might indicate a mismatch between the abstraction hierarchy and the characteristics of the system to be mapped and the typical tasks participants perform within the system. Future studies should therefore examine which representational frameworks are suitable to assess functional understanding for different types of systems.

Regarding concept map contents, participants used more functional and fewer non-functional propositions (e.g., structural or temporal propositions) with concept mapping. A closer look into the functional propositions (i.e., whether they were correct, false, or reversed the direction of the relationship) did not reveal differences between concept mapping and verbal explanation. In absolute terms, however, participants included more correct functional propositions when using concept mapping. Thus, the content-related quality was higher for participant-constructed concept maps. From a more practical perspective, it should be noted that participants needed much more time to explain system functioning in a concept map. However, this disadvantage can be compensated for by testing multiple people at once, which is not possible for verbal explanation. Taken together, since participants produced better explanations of system functioning for well-known systems, the proposed concept mapping method seems to be the more appropriate for assessing functional understanding.

In the following sections, we will discuss potential reasons for the observed benefit of concept mapping, and how the method could be adapted to assess understanding of more complex systems. We will also address the limitations of the present study and use them to derive perspectives for future research.

### 4.1. Why Does Concept Mapping Lead to Better Explanations of System Functioning?

When externalizing their understanding of system functioning, participants must perform two steps: they must explicate the relation between the concepts, and they must structure their explanation (e.g., decide when to use which concept for explaining system functioning). We argue that the demands posed by these steps differ between the assessment methods, because graphical representation and verbal explanation differ in two important ways, namely regarding the extent to which a syntactic structure is pre-defined and regarding the extent to which organization processes are supported.

For concept mapping, the syntactical structure is clearly defined (concept–relation–concept), and participants must translate their understanding to fit this structure. Concept mapping forces participants to specify the relations between concepts as much as possible [19,21], while at the same time supporting the required organizing processes [34]. When explaining system functioning verbally, however, organizing processes are not supported. Instead, participants must remember which concepts they have already used and which relations still need explaining. On the other hand, verbal explanation offers more flexibility. For example, participants may connect more than two concepts in one statement. Since verbal explanations were also transformed into concept maps for the analysis of individual concept–relation–concept units, such elaborate explanations were chopped up into segments that did not make sense anymore on their own. Consequently, the benefit of concept mapping over verbal explanation is closely linked to the coding and scoring of participants’ explanations, which was based on isolated propositions in the present study.

Regarding the use of concept maps in practical settings such as educational contexts, our results suggest that following a short instruction, people can express their understanding in terms of a concept map. However, the produced concept maps were far from perfect, even though the systems to be mapped were simple, everyday systems. Research on different types of concept maps has identified several strategies for increasing map quality. For example, map quality is higher when participants carefully plan the construction process [19] or when they are instructed to only include necessary links and focus on the temporal flow of causal relations [32]. Future studies could examine whether these strategies are also helpful for constructing high-quality maps that specifically focus on functional relations. These strategies should subsequently be included in concept mapping instructions to help participants construct accurate representations of their understanding.

### 4.2. How Should the Concept Mapping Method Be Adapted to Assess Understanding of Complex Systems?

In the present study, participants were asked to explain the functioning of two simple, everyday systems (i.e., a bike and sink). The reference maps used to judge participants’ explanations consisted of 14 propositions that connected 13 and 14 concepts for the bike and the sink, respectively. In subsequent research [36], we applied the graphical representation method presented here and found that the resulting concept maps could be used to assess understanding of more complex systems (i.e., fuel temperature control using 20–22 concepts). However, as complexity increases further, systems are characterized by a larger number of variables that are connected in multiple, sometimes intransparent ways, and that may change dynamically [37]. Our findings suggest that if the understanding of more complex systems is to be assessed with concept maps, the method should be adapted in two ways.

First, the task as well as the coding procedure should be adapted to allow for relations between more than two concepts. In the present study, we instructed participants to explain system functioning by describing the relation between two specific concepts. As system complexity increases, it is expected that functional relations include more than two concepts. While in principle, such relations can be represented in a concept map, participants in our study did not make use of this possibility. During verbal explanation, on the other hand, participants frequently connected more than two concepts within one sentence. For example, one participant verbally explained the consequences of different sprocket sizes for how fast the wheels of the bike were turning. Thus, participants might need explicit instructions on how to connect more than two concepts in their maps. It should also be noted that judging such structures is not trivial, either. In the present study, coding was based on propositions, namely individual concept–relation–concept entities. For the participant introduced above, this meant that the elaborate explanation was chopped up into segments that did not make sense anymore on their own and consequently, we likely underestimated his actual understanding of system functioning. Therefore, a coding protocol would be required that can be applied to concept map structures spanning more than two concepts.

Second, participants should have the option to make small changes to the provided material. In the present study, participants were not allowed to add concepts or change the wording, but could only specify the relations between the provided concepts or decide not to use some concepts at all. In the graphical condition, these rules were followed, but in the verbal condition, we observed that participants interacted more flexibly with the provided concepts; for example, by changing the formulation so that it fit their explanation better. Thus, it seems that the provided material posed rigid boundaries for explaining system functioning graphically, but not verbally. Of course, increasing the degrees of freedom for interacting with the provided materials may reduce the comparability of explanations across participants. On the other hand, it also increases the chance that participants can compensate for ambiguity in the provided material, thereby allowing them to really express *their own* understanding of system functioning. This is especially relevant for more complex systems, as perspectives on the same system can differ greatly between experts [16]. If several perspectives on system functioning are valid, participants should be able to make changes to the provided material.

### 4.3. Limiations and Perspectives for Future Research

When considering our results, some limitations should be kept in mind. First, we have defined system functioning according to the abstraction hierarchy, consisting of the purposes, functions, components, and properties of a system that are connected via means–ends links. Consequently, the benefits and drawbacks of concept mapping compared to verbal explanation relate specifically to this framework. However, several other methods are available to represent system functioning, such as the Functional Resonance Analysis Method (FRAM) for modeling functional dependencies within a system [38]. These methods may also be more appropriate for modeling highly complex systems. Future research should examine how different functional frameworks can be used to assess understanding of system functioning and how domain and task requirements influence which methods fit the given context best.

Second, participants in the present study were instructed to explain system functioning according to an abstraction hierarchy, independent of whether they used concept mapping or verbal explanation. Nevertheless, participants seemed to adhere to the instructions more closely during concept mapping. This raises the question whether participants can better express their understanding of system functioning with concept maps, or whether concept mapping simply helps them to adhere to the instructions and focus on the task, whatever that task may be. In the future, this question could be addressed by manipulating the instructions and testing the respective effects. For example, the concept mapping instruction could explicitly introduce links other than means–ends relations. Conversely, participants could be instructed to plan their verbal explanations more carefully and focus specifically on means–ends relations. This should indicate whether the differences between concept mapping and verbal explanation actually depend on the mode of externalization, rather than on the degrees of freedom afforded.

Third, our sample consisted exclusively of German-speaking participants. Thus, the benefit of graphical representation over verbal explanation can only be established for German. For languages following a different syntactical structure, the propositional structure of concept maps might be less suitable. One study with Turkish participants reported difficulties in transferring Turkish sentences into propositions, as these sentences do not make sense when transformed into subject–verb–object propositions [39]. Thus, comparisons between concept maps and verbal explanation for syntactically different languages should be conducted to examine the generalizability of our findings across languages.

Another limitation pertains to skills and abilities besides functional system knowledge that might influence concept map quality. We have previously shown that people who know more about system functioning also produce concept maps with higher content-related quality [36]. In the future, additional factors influencing understanding should be examined to shed light on the relationship between functional understanding and concept map quality. For example, it would be interesting to examine how intelligence or previous experience with functional thinking influence concept map quality.

Finally, the present study cannot answer how functional understanding as defined here relates to the use of this understanding to solve real-world problems. Research on complex problem solving has found that in general, people who know about cause-and-effect relations within a system show better performance when controlling that system (e.g., [40]). Future research should examine how people use what they know about system functioning for specific tasks, such as formulating hypotheses about potential causes for deviating system behavior, or planning how to test these hypotheses.

## 5. Conclusions

Graphical representation and verbal explanation displayed complementary benefits and drawbacks for explaining system functioning: participants described more functional relations when concept mapping, and these relations were no less accurate than those produced through verbal explanation. However, concept maps seem to focus participants’ attention on associations between pairs of concepts, which may make them less suitable to represent more complex relations. Concept mapping further requires training and takes longer to complete. Verbal explanation, on the other hand, is much faster and well suited to depict relations between more than two concepts. However, participants differ greatly in their approaches to verbal explanation, and they may change the provided concepts to better fit what they are trying to say. This makes verbal explanations less comparable and harder to rate consistently. In sum, concept mapping seems to be especially useful for large-scale assessments, and for less complex systems for which a single valid perspective on system functioning can be defined. For more complex systems, relations other than means–ends relations should be included, and participants should be allowed to adapt the provided material to depict their specialized views on system functioning.

## Figures and Tables

**Table 1:** Descriptive statistics and *t*-test results for time on task, propositions, correct functional propositions, and intersection over union.

Dependent Variables	Graphical		Verbal		*t*	*p*	Cohen’s *d*
	*M*	*SD*	*M*	*SD*			
Time on task (min:s)	17:07	5:03	3:47	1:54	11.59 ^a^	<0.001	2.47
Propositions	14.0	1.6	15.9	3.2	−2.82 ^b^	0.010	−0.58
Correct functional propositions	12.1	2.5	9.3	3.0	3.87 ^b^	<0.001	0.79
Intersection over union	0.49	0.19	0.41	0.18	1.60 ^b^	0.123	0.33

^a^ *t*(21). ^b^ *t*(23).

**Table 2:** Descriptive statistics for the proportion of different proposition types.

Proposition Types	Graphical		Verbal	
	*M*	*SD*	*M*	*SD*
Functional	97.4	7.1	64.6	19.0
Correct	89.0	13.5	92.2	10.0
Wrong direction	6.4	8.3	0.6	2.2
False	4.6	7.5	7.1	10.0
Structural	0.3	1.3	9.9	10.6
Temporal	0	0	3.7	7.5
General	2.3	6.6	17.5	12.3

## Data Availability

All study materials as well as the data and analyses were made available via the Open Science Framework: https://osf.io/kneah/.
